# Selective Bacterial Community Enrichment between the Pitcher Plants Sarracenia minor and Sarracenia flava

**DOI:** 10.1128/Spectrum.00696-21

**Published:** 2021-11-24

**Authors:** Scott M. Yourstone, Ilon Weinstein, Elizabeth Ademski, Elizabeth A. Shank, Nikolas M. Stasulli

**Affiliations:** a Curriculum in Bioinformatics and Computational Biology, University of North Carolina, Chapel Hill, North Carolina, USA; b Department of Biology, University of North Carolina, Chapel Hill, North Carolina, USA; c Program in Systems Biology, University of Massachusetts Medical School, Worcester, Massachusetts, USA; d Department of Microbiology and Physiological Systems, University of Massachusetts Medical School, Worcester, Massachusetts, USA; e Department of Biology and Environmental Science, University of New Haven, West Haven, Connecticut, USA; USDA—San Joaquin Valley Agricultural Sciences Center

**Keywords:** microbial communities, microbiome, bacterial genomics, pitcher plants, *Sarracenia*

## Abstract

The interconnected and overlapping habitats present in natural ecosystems remain a challenge in determining the forces driving microbial community composition. The cuplike leaf structures of some carnivorous plants, including those of the family *Sarraceniaceae*, are self-contained ecological habitats that represent systems for exploring such microbial ecology questions. We investigated whether Sarracenia minor and Sarracenia flava cultivate distinct bacterial communities when sampled at the same geographic location and time. This sampling strategy eliminates many abiotic environmental variables present in other studies that compare samples harvested over time, and it could reveal biotic factors driving the selection of microbes. DNA extracted from the decomposing detritus trapped in each *Sarracenia* leaf pitcher was profiled using 16S rRNA amplicon sequencing. We identified a surprising amount of bacterial diversity within each pitcher, but we also discovered bacteria whose abundance was specifically enriched in one of the two *Sarracenia* species. These differences in bacterial community representation suggest some biotic influence of the *Sarracenia* plant on the bacterial composition of their pitchers. Overall, our results suggest that bacterial selection due to factors other than geographic location, weather, or prey availability is occurring within the pitchers of these two closely related plant species. This indicates that specific characteristics of *S. minor* and *S. flava* may play a role in fostering distinct bacterial communities. These confined, naturally occurring microbial ecosystems within *Sarracenia* pitchers may provide model systems to answer important questions about the drivers of microbial community composition, succession, and response to environmental perturbations.

**IMPORTANCE** This study uses amplicon sequencing to compare the bacterial communities of environmental samples from the detritus of the leaf cavities of Sarracenia minor and Sarracenia flava pitcher plants. We sampled the detritus at the same time and in the same geographic location, eliminating many environmental variables present in other comparative studies. This study revealed that different species of *Sarracenia* contain distinct bacterial members within their pitchers, suggesting that these communities are not randomly established based on environmental factors and the prey pool but are potentially enriched for by the plants’ chemical or physical environment. This study of these naturally occurring, confined microbial ecosystems will help further establish carnivorous pitcher plants as a model system for answering important questions about the development and succession of microbial communities.

## INTRODUCTION

A central question in the field of microbial ecology is how specific environments deterministically shape their microbial communities (1). One challenge has been that most natural habitats are spatially continuous, often leading to dynamic mixing of adjacent communities. Systems comprised of naturally established yet spatially defined microbial communities are therefore valuable for addressing the question of how natural habitats influence microbial community composition. Phytotelmata (compartments of terrestrial plants that collect and retain rainwater) provide ideal systems for interrogating such self-contained microbial communities (2). One group of phytotelmata that have a functional purpose are those formed by the pitfall traps of carnivorous plants of the family *Sarraceniaceae* (3, 4). These plants are therefore a natural system well suited to studying the potential effects that plants or founder microbes may have on the establishment of a microbial community.

Previous *Sarracenia* pitcher plant microbiome studies have focused on a range of temporal (5–8) and geographic (7, 9–11) questions. To date, only one study (12) has investigated whether *Sarracenia* microbiomes are more impacted by plant-specific features (such as biochemical secretions or attracted prey) or geographic location (including environmental factors and their impact on prey pools). This study seeks to further explore this question by comparing the pitcher microbiota of two related *Sarracenia* species that were present in the same geographic location using samples collected at the same time; thus, weather could not influence the microbiota between samplings. This is a critical question because the source of bacteria within pitcher plant leaf cavities has not been clearly established. It has been proposed that *Sarracenia* pitchers are sterile prior to opening based on the results of bacterial culturing efforts, culture-independent PCR assays, and microscopy (13, 14). If true, the resident microbiota of the prey themselves may act as a source of the pitcher plant’s microbiota; thus, the geographically defined prey pools accessible to *Sarracenia* plants could strongly influence their resulting microbiota. In addition, there are also phenotypic and chemical differences between different *Sarracenia* plant species (3, 15, 16) that could influence prey attraction and microbial survival within the pitchers. To begin to address this question, we examined the bacterial communities present within the pitchers of two *Sarracenia* species (Sarracenia minor and Sarracenia flava) grown in the same geographic location and sampled at the same time.

**FIG 1:**
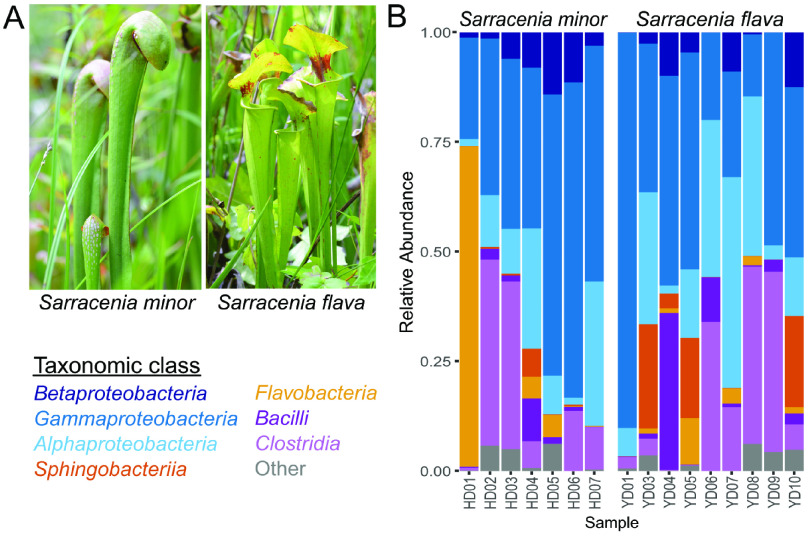
Initial sampling and community profiling of pitcher plant detritus. After sampling the detritus of both (A) *Sarracenia minor* (left) and *Sarracenia flava* (right), the bacterial communities were profiled using the 16S rRNA gene amplicon sequencing with the number of reads collected from each sample ranging between 1 × 10^5^ and 8 × 10^5^ reads per pitcher. The 16S rRNA reads were then compared to known genetic sequences to determine (B) the relative abundance of different bacterial phylogenetic classes within each pitcher.

*S. minor* and *S. flava* are phylogenetically related but structurally distinct plants: *S. minor* (the hooded pitcher plant) has an operculum that folds forward over the front of the pitcher opening and closes off much of the pitcher to the surrounding environment, while *S. flava* (the yellow pitcher plant) has an operculum that is raised above the pitcher opening, leaving it more open to the environment (see Fig. 1A). Using 16S rRNA gene profiling, we analyzed the bacterial communities of *S. minor* and *S. flava* plants grown in a natural setting in the same location. This sampling strategy removed confounding temporal and geographic variables, thus mitigating the influence of differences in abiotic environmental factors or potential overall prey availability on the bacterial community composition of these two *Sarracenia* pitchers and enabling us to attribute observed differences to plant species-specific factors. As described below, our results indicate that these two plant species do, either directly or indirectly, enrich for different bacterial community members within their pitchers. These results provide a foundation to begin questioning the plant-specific factors, physical or chemical, that may impact the bacterial communities of these closely related plant species.

## RESULTS

The goal of this study was to investigate the bacterial community composition of the leaf cavities of two species of pitcher plant using a sampling structure that reduced as many abiotic influences as possible (such as weather changes or seasonal prey availability). Based on the number of samples that could be collected and processed on the same day, this approach led to a relatively small number of *Sarracenia* samples being utilized here. The detritus from nine pitchers of *S. flava* and seven pitchers of *S. minor* (Fig. 1A) was assessed for bacterial community analysis using 16S rRNA gene sequencing (Table 1). We elected not to normalize the quantity of detritus across plants in order to maximize DNA extraction yields and therefore obtained varying numbers of sequencing reads, depending on the pitcher sample (Fig. S1). When assigning these reads to operational taxonomic units (OTUs), we chose a relatively conservative cutoff, only considering those OTUs that were represented by at least 25 sequence reads in at least two separate samples; this eliminated many singleton reads (Fig. S2) and resulted in 644 OTUs (representing 89% of the total 6,068,766 reads obtained). The overall phylogenetic composition of the bacterial communities within the pitchers’ detritus included bacteria from eight dominant phyla (representing 98.6% of OTUs), with *Bacteroidetes*, *Firmicutes*, and *Proteobacteria* being the most abundant (Fig. 1B).

**TABLE 1:** Weight of *Sarracenia* sp. detritus collected for genomic isolation using a Qiagen PowerSoil DNA isolation kit

Data for Sarracenia minor:		Data for Sarracenia flava:	
Plant identifier	Wt (g)	Plant identifier	Wt (g)
HD01	0.0482	YD01	0.1000
HD02	0.1045	YD03	0.2457
HD03	0.0212	YD04	0.1408
HD04	0.1807	YD05	0.2418
HD05	0.0233	YD06	0.2432
HD06	0.1067	YD07	0.2143
HD07	0.0323	YD08	0.2344
		YD09	0.0146
		YD10	0.2405

We then examined the bacterial diversity present in the samples from these two plant species. Calculations of taxonomic diversity on rarefied data using the phylogenetic diversity (PD) whole-tree alpha diversity metric indicated that there were no significant differences in alpha diversity between the bacterial communities of these plant species (Fig. S3). Beta diversity, via the weighted Bray-Curtis dissimilarity metric, was incorporated into a canonical analysis of principal coordinates (CAP) model that was constrained by plant species. We elected to use this approach since all samples were harvested on the same day from the same location, and we were specifically interested in determining whether there were bacterial community differences dictated by the plant species. This CAP model indicated that approximately 10.2% of the variance between samples was attributable to the plant species alone (Fig. S4).

**FIG 2:**
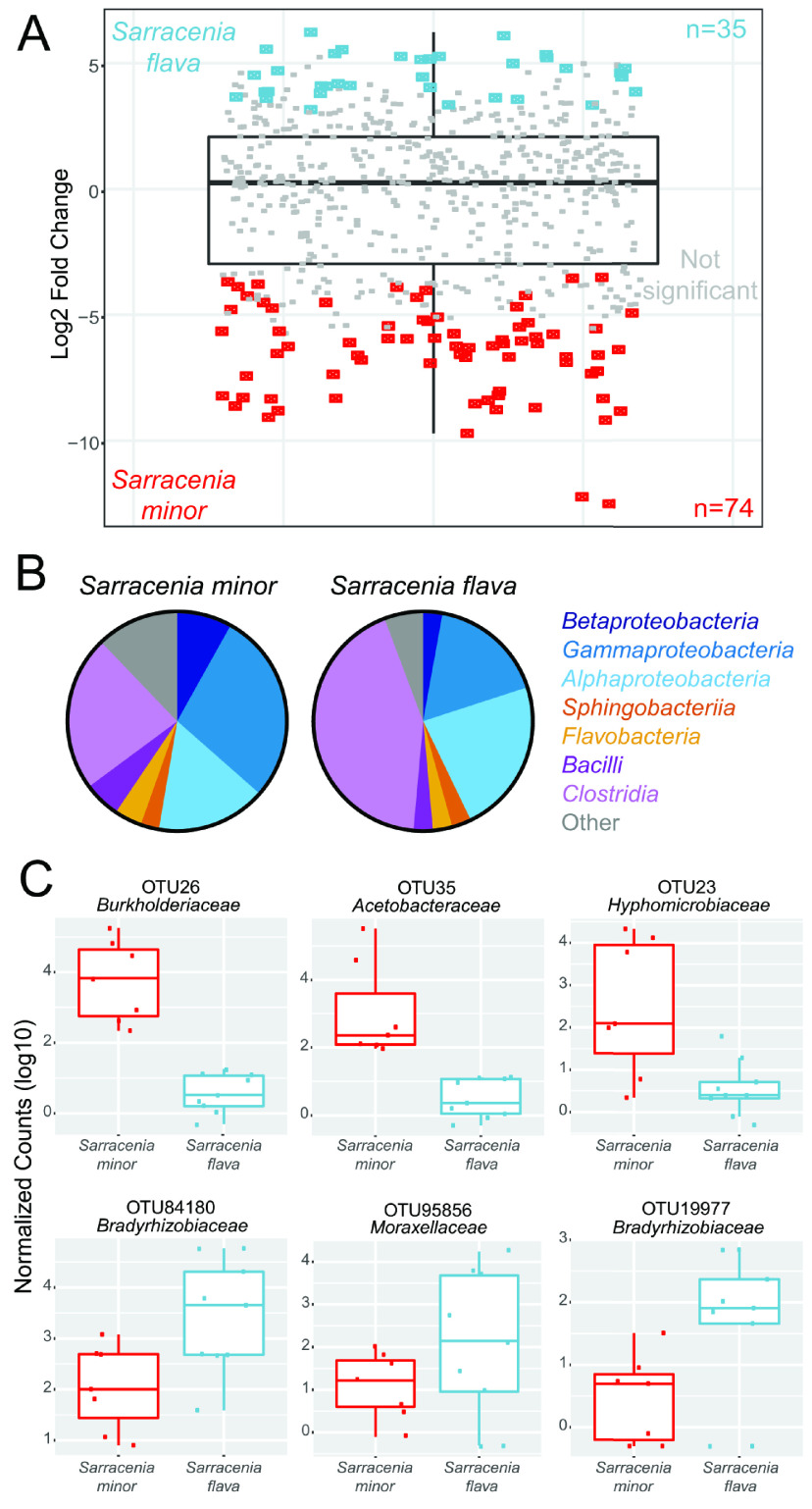
Identification of differentially expressed OTUs using a negative binomial generalized linear model. Using this model, we (A) identified the OTUs within our two pitcher plant species that are differentially expressed (α < 0.05), while also considering the number of samples in which they are represented. (B) Pie charts of the differentially expressed OTUs indicate that *Gammaproteobacteria* are enriched across most of the *S. minor* pitchers, while *Alphaproteobacteria* and *Clostridia* are enriched across a large portion of the *S. flava* pitchers. (C) Representative normalized count box plots of some of the most differentially expressed OTUs (with their taxonomic family) visually confirms that these OTUs are enriched between the two pitcher plant species.

We next identified specific OTUs that were differentially abundant between the two pitcher plant species. To do so, we built a negative binomial generalized linear model using DESeq2, which takes into account differences in sequencing depth between samples. This model identified 35 OTUs in *S. flava* and 74 OTUs in *S. minor* that were differentially enriched at a significance threshold of α < 0.05 (Fig. 2A). For both plant species, the enriched OTUs most frequently fell within the classes *Gammaproteobacteria*, *Alphaproteobacteria*, and *Clostridia* (Fig. 2B); these are the same bacterial classes that were most abundant in the overall pitcher plant communities (Fig. 1B).

Due to the variability observed in bacterial community composition across samples—even those from the same plant species—we next examined how individual enriched OTUs were distributed across each pitcher sample. Figure 2C shows the distributions of representative enriched OTUs and their normalized counts within each sampled pitcher. These data demonstrate that enriched OTUs are indeed more abundant across the majority of samples from a single plant species (Fig. 2C). While different bacterial clades were enriched in the two plant species, no single phylogenetic class showed enrichment in only one of the pitcher plant species (Fig. 2B).

## DISCUSSION

Our results indicate that *Sarracenia* carnivorous pitcher plant species can harbor distinct bacterial community members unrelated to geographic location, weather, or prey availability. These data suggest that other biotic factors specific to the plant species (such as prey attraction, chemical secretions in or around the opening of the pitcher, or differences in the plant and insect litter present inside the leaf structure) may drive the enrichment of specific bacterial groups within pitcher leaf cavities. These findings facilitate the generation of additional hypotheses regarding the factors influencing microbial succession and diversity between these plant species and about how the enriched clades of bacteria may influence the function of the bacterial communities they inhabit.

Due to pitcher plants housing decomposing insects inside their cupped leaf structure, their microbiome has often been hypothesized to act as a plant “gut,” digesting prey to provide nutrients to the plant. It was previously suggested that there is similarity at the phylum level between the microbiome of pitcher plants and the human gut (10). However, when these claims were tested, it was determined that pitcher plants have a microbiome profile distinct from the human gut, the mouse gut, the phyllosphere of Arabidopsis thaliana, and the soil (17). Consistent with these findings, if we compare our results to recent reviews summarizing the microbiome composition of the phyllosphere (18) and 40 different host species of plant and animals (19), we observe that the microbiomes of *S. flava* and *S. minor* are not clearly aligned with either human gut or plant communities but lie somewhere in between. Almost all our pitcher plant samples are dominated by proteobacteria (specifically *Alphaproteobacteria* and *Gammaproteobacteria*) (Fig. 1B), clades that are highly represented in rhizosphere and phyllosphere microbiomes (18, 19), while the next-most-abundant group in our *Sarracenia* samples, the *Clostridia* clade of *Firmicutes* (Fig. 1B), is most highly represented in animal gut microbiomes. The third-most-abundant clade in our data set, the *Bacteroidetes* (Fig. 1B), is present in both rhizosphere and gut communities, with its abundance depending on the particular bacterial class examined (18, 19). Our data are thus consistent with the idea that the microbiomes of *S. flava* and *S. minor* pitchers reflect features of both plant- and gut-associated communities.

Our approach of collecting all samples on the same day eliminated many challenging-to-control abiotic variables. However, one factor that we did not explicitly consider was the successional stage of each plant: we do not know how long each pitcher was open prior to sampling. In addition, the time between pitcher opening and initial prey capture, as well as which insects were trapped and in what order, may have varied substantially across different pitchers. Indeed, visual inspection indicated that the insects inside the pitcher were at different stages of decomposition, suggesting that they had been open for different periods of time prior to collection. Despite being unable to account for the timing of prey accumulation and successional stage, our results still indicate the enrichment of specific bacterial community members in the *S. flava* and *S. minor* pitcher samples (Fig. 2).

Several speculative hypotheses exist as to how these two *Sarracenia* species might generate these distinct bacterial communities. One is that the different *Sarracenia* (Fig. 1A) could lure or trap different prey based on their physiology (15, 20–23). This might directly impact the resulting plant microbiomes based on the bacteria associated with the incoming prey. Ants and other Hymenoptera are the insects most commonly attracted to *Sarracenia* pitcher plants (3, 24, 25); *S. minor*, specifically, seems to be an ant specialist (26, 27). However, the microbiomes of ants tend to vary depending on their geographic location (28–30). If geography also impacts the microbiome of other insects, this could explain the differences previously observed in bacterial community composition between geographically distinct *Sarracenia* populations. Because we lack environmental insect specimens from the same location where our pitcher plants were sampled, it is impossible to deduce whether a difference in prey-associated microbes may be driving the differences we observed between the *S. flava* and *S. minor* bacterial communities. Anecdotally, we can report that one of the *S. minor* pitchers did contain a small frog that had begun to decompose. We postulate that this frog may have impacted the alpha diversity of that pitcher sample (it was the sample with the greatest observed diversity; Fig. S3), and we predict that prey capture may have had a substantial effect on the range of alpha diversity we observed.

Another influence on bacterial community composition and succession could be any inquiline communities that exist in the pitchers of these two *Sarracenia* species. Other than *S. flava* being an obligate host of the Exyra ridingsii moth (31), there is limited evidence of inquiline communities being present in *S. flava* or *S. minor* (3, 31). However, the related species Sarracenia purpurea is known to contain extensive inquiline communities (3, 32–40) that affect the pitcher’s microbial communities through predation (14, 41). It is therefore likely that if inquiline food webs exist within *S. minor* or *S. flava*, they could have an impact on the microbial communities present in their pitchers. The different hood structures of these plants (Fig. 1A) could also affect moisture levels inside the pitchers, which could influence the establishment of any inquiline communities within them and the resulting microbial communities as well (3).

Another explanation for the enriched abundance of different bacterial clades between *S. flava* and *S. minor* is that the plants could be actively selecting for certain bacterial members through chemical secretions or pH changes, as has been detected in *Nepenthaceae* pitcher plants (42–44). Although no *Sarracenia* species has been shown to actively influence their microbiota, *Sarracenia purpurea*, a plant used medicinally by the Canadian First Nations, produces antimycobacterial compounds (45), indicating that *Sarracenia* produces chemical cues which may have the capacity to influence microbial survival in their pitchers. Additionally, a large percentage of *Sarracenia* species secrete proteases that may help decompose insects (46) and, in turn, could influence the nutrients available to the microbial communities. A recent study using gas chromatography-mass spectrometry to look at the metabolites of multiple pitcher plant species noted differences among the many plant accessions examined (including those of *S. flava*, *S. minor*, and six other *Sarracenia* species), although the metabolic data does not fully correspond with plant phylogeny (16).

Overall, this study reveals that distinct enriched bacterial OTUs are present in *S. flava* and *S. minor* pitchers that were sampled at the same time and geographical location. This unique sampling approach represents a valuable way to obtain insights about the interrelated biotic and abiotic factors impacting the microbial composition of these physically constrained pitcher leaf environments. Future work will help us better understand the factors driving the establishment and maintenance of these microbial communities. For instance, studies detailing the insect species being lured and the natural rainfall or passive influx of debris into the plant could provide insights regarding how these communities are established, while characterizing the chemical compounds secreted by these *Sarracenia* species, and the influences they have on bacterial community members, will provide information as to the plant’s biochemical influences on their pitcher microbiomes. Additionally, manipulative studies altering the opercula of different *Sarracenia* plants could provide useful information as to the influence of physical features of the *Sarracenia* plant structures.

## MATERIALS AND METHODS

### Pitcher plant collection and detritus extraction.

In collaboration with the North Carolina Botanical Gardens, we were given access to a semicurated, employees-only bog garden where *S. flava* and *S. minor* were both growing adjacent to one another. In this area, individual *Sarracenia* species’ rhizomes had been separated into pots and placed in a sunken bog and were periodically maintained and propagated by the NC Botanical Gardens. On 17 June 2016, we identified mature pitcher leaves that had opened that season and were actively capturing prey; we selected leaves that did not have any damage to the length of the pitcher and did not have their cup opening obstructed by other bog debris such as leaves or twigs. The pitchers of the same species were intentionally not sampled from the same pot or rhizome, but some could potentially be clonal pitchers where the rhizomes had been split in previous years. These sampling parameters informed much of our sampling strategy and limited the number of pitchers we were able to sample. Based on these restrictions, we collected seven *S. minor* pitchers with average heights of 41.6 ± 3.8 cm and 10 *S. flava* pitchers with average heights of 48.3 ± 10.5 cm. The collected pitchers were no more than 1 m from each other, and all pitchers were collected from an approximate 2.4 × 3.6-m area. Pots of the two *Sarracenia* species were primarily clustered together; there were other plant species and *Sarracenia* hybrids present in the area as well. Each pitcher was severed from the plant at ground level. The pitchers were collected in sterile Whirl-Pak sample bags and placed on ice in a cooler for transport. The pitchers were stored on ice for no more than 2 h before processing. To decrease any potential contamination of the inside of the pitcher during dissection, out of an abundance of caution, we disinfected the outside of each pitcher twice using an autoclaved-sterilized cotton swab saturated with 70% ethanol and allowed it to air dry. One side of each pitcher was sliced vertically using a new, sterile scalpel, and the detritus at the very bottom of the cuplike leaf structure, where the decomposing organic matter had settled over time, was collected and weighed (see Table 1). Information on the species diversity or numbers of captured prey was not collected: for some pitchers, all of the available detritus was used for DNA extraction, and thus any inquiline study would have been incomplete.

### Bacterial community profiling of pitchers.

The detritus was weighed (Table 1), and the entire quantity collected was immediately processed using the PowerSoil DNA isolation kit (Qiagen). The resulting DNA was sent to the UNC—Chapel Hill High-Throughput Sequencing Facility for paired-end 16S rRNA gene sequencing on an Illumina MiSeq instrument using a previously described molecular-tagging protocol (47) with the 515F and 806R 16S rRNA gene primers covering the V4 region. Although 10 *S. flava* pitchers were sampled, one of the library preparations failed and had to be removed from the analysis. The sequence data have been submitted to GenBank under BioProject accession number PRJEB22641. MT-Toolbox (48) was used to remove the molecular tags from the sequencing reads as well as to remove low-quality reads based on the program’s default settings.

### Identifying operational taxonomic units.

Using a 97% identity cutoff and filtering of chimeric sequences, high-quality reads were clustered into 98,584 OTUs using open reference picking with version 7.0.1090 of the USEARCH algorithm (49), as implemented in the metagenomics plugin of MT-Toolbox (48). Chloroplast and mitochondrial sequences were removed using BLAST. OTUs with fewer than 25 reads in at least two samples were removed; this was a conservative cutoff to minimize false positives in our analyses and resulted in 644 measurable OTUs. This set of OTUs contains 89% of the total high-quality sequence reads obtained (6,071,825 out of 6,849,737) and was used for all further analysis. Taxonomic assignments were made for each OTU using the assign_taxonomy.py script implemented in QIIME (50), in conjunction with the May 2013 version of the Greengenes database (51).

### Custom analysis scripts.

Analyses were performed on nonrarefied data, except for the alpha diversity calculations. All custom scripts are accessible via GitHub at https://github.com/islandhopper81/pitcher_plant_utils. The names of specific scripts used in our analyses are noted in parentheses below.

### Identification of enriched OTUs using DESeq2.

The DESeq2 library (52) was used to call OTUs enriched in either plant species. DESeq2 models OTU read counts using a negative binomial distribution and is a tool commonly used to identify condition-specific OTUs. DESeq2 accounts for differences in sequencing depth between samples (52). Our model included the plant species as the only factor. Custom scripts were used to streamline this process (model_main.R, make_tax_table.pl, and make_otu_boxplots.R). For this analysis, OTUs were analyzed based on the plant species they came from. The analysis considered both the number of reads of that OTU (represented as a fraction of reads in *S. flava* samples compared to the reads in *S. minor* samples) and the number of plants per species in which the OTU was present. The DESeq2 data set was created from the OTU count data matrix generated from the 16S rRNA sequencing reads. The metadata file was also read in as a matrix, and the plant species column was selected as the parameter around which the negative binomial distribution was calculated. All other parameters remained set to the DESeq2 defaults, and the OTUs identified as enriched are based off a differential abundance with a significance of α < 0.05.

### Beta diversity.

A custom script (cap_main.R), presenting a canonical analysis of principal coordinates (CAP) model (53, 54), utilizing the vegan package (55) capscale function was used to calculate the beta diversity between samples. For this CAP analysis, the weighted Bray-Curtis dissimilarity metric was used, and the analysis was constrained by the plant species metadata metric. This allowed us to identify the variance in the microbiome community composition that could be accounted for by the *Sarracenia* species from which the sample was collected.

### Alpha diversity.

The alpha diversity for each sample was calculated with rarefied data (Fig. S5) using the PD whole tree, Chao1, Shannon, and Simpson metrics as implemented in the QIIME script alpha_diversity.py (50). Student’s *t* test was used to test if PD whole tree numbers differed between the two plant species (custom script make_alpha_div_fig.R). The Chao1 analysis, however, was not considered due to the filtering steps described above, since this analysis can be affected by the removal of rare or singleton reads (56).

### Data availability.

The data generated and analyzed during this study have been submitted to the NCBI GenBank database under BioProject accession number PRJEB22641.
