# Supplementary material for: Selective Bacterial Community Enrichment between the Pitcher Plants Sarracenia minor and Sarracenia flava
Source: Microbiol Spectr. 2021 Nov 24;9(3):e00696-21. doi: 10.1128/Spectrum.00696-21 (PMC8612160; doi:10.1128/Spectrum.00696-21)
Supplement: SUPPLEMENTAL FILE 1 — Supplemental material. Download SPECTRUM00696-21_Supp_1_seq6.pdf, PDF file, 0.6 MB [file spectrum00696-21_supp_1_seq6.pdf]

SUPPLEMENTAL MATERIALS

Figure S1 - .pdf – Figure_S1_read_counts – a graphical representation of the read counts that were acquired for each of the samples analyzed.

Figure S2 - .pdf - Figure_S2_otu_filtering – a graphical representation of the OTU thresholding used for conservatively filtering the OTU data.

Figure S3 - .pdf – Figure_S3_alpha_div_test – a graphical representation of the alpha diversity calculation on data rarefied to 50,000 reads comparing the values of each microbial community between the two plant species.

Figure S4 - .pdf – Figure_S4_CAP_analysis – a graphical representation of a canonical analysis of principal coordinates (CAP) analysis that was constrained by plant species, including Beta diversity calculations using the weighted Bray-Curtis dissimilarity metric.

Figure S5 - .pdf – Figure_S5_rarefaction – a graphical representation of various rarefaction thresholds and our data represented within those thresholds.

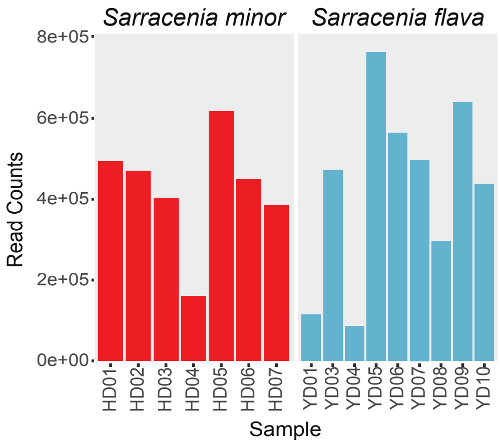
Sarracenia minor
Sarracenia flava
Read Counts
8e+05
6e+05
4e+05
2e+05
0e+00
HD01
HD02
HD03
HD04
HD05
HD06
HD07
YD01
YD03
YD04
YD05
YD06
YD07
YD08
YD09
YD10
Sample

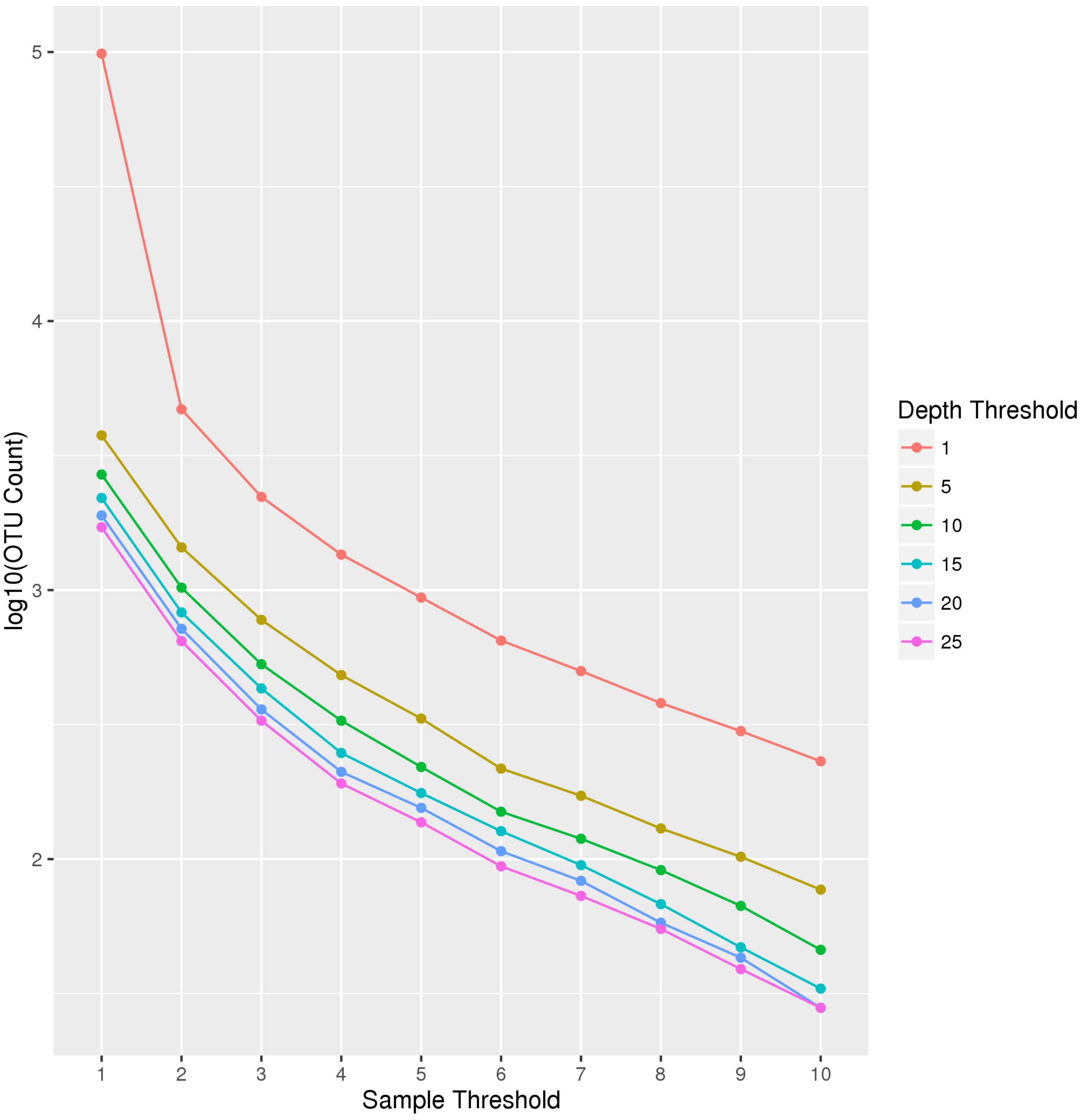

Depth Threshold
1
5
10
15
20
25
log10(OTU Count)
5
4
3
2
1
2
3
4
5
6
7
8
9
10
Sample Threshold

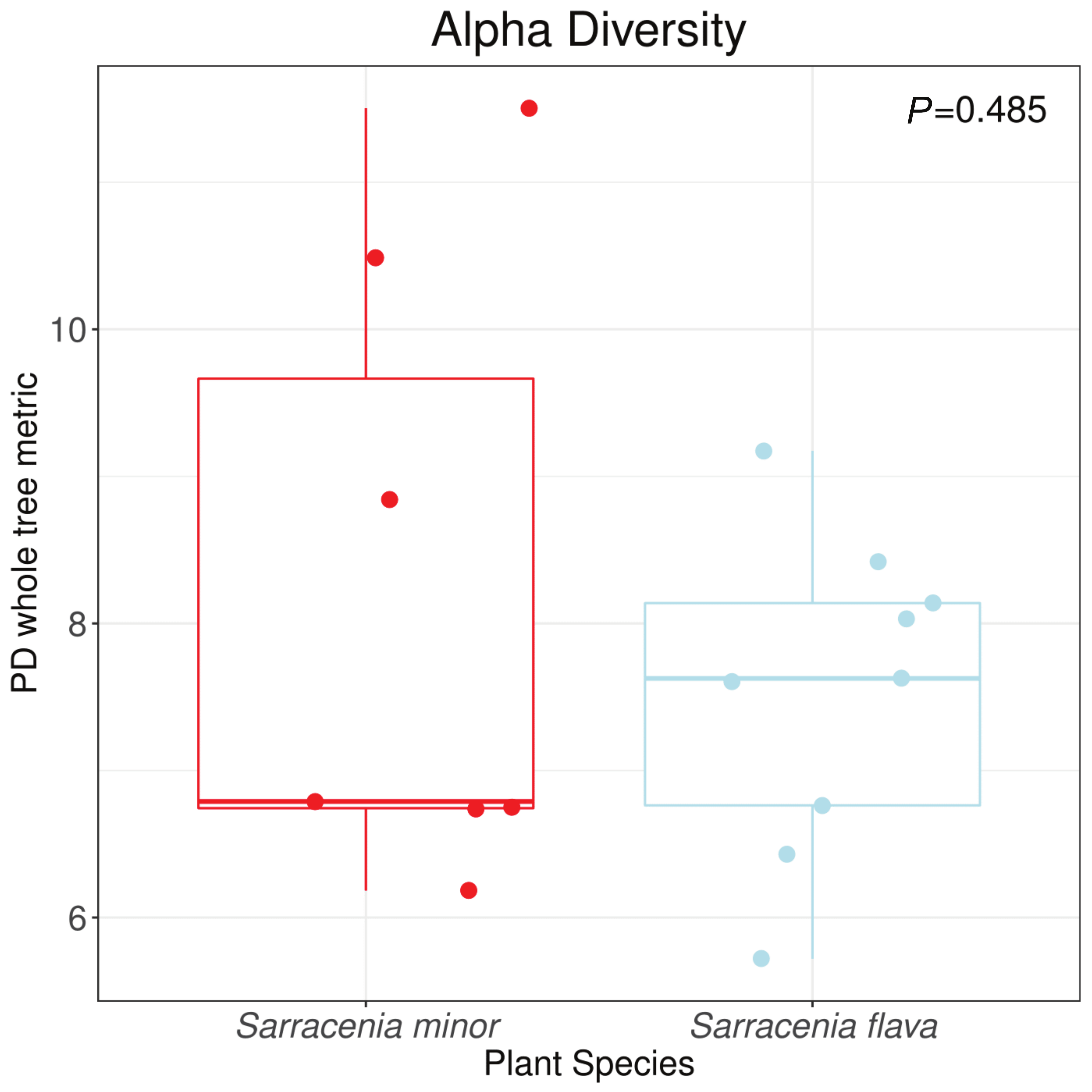
Alpha Diversity
P=0.485
PD whole tree metric
10
8
6
Sarracenia minor
Sarracenia flava
Plant Species

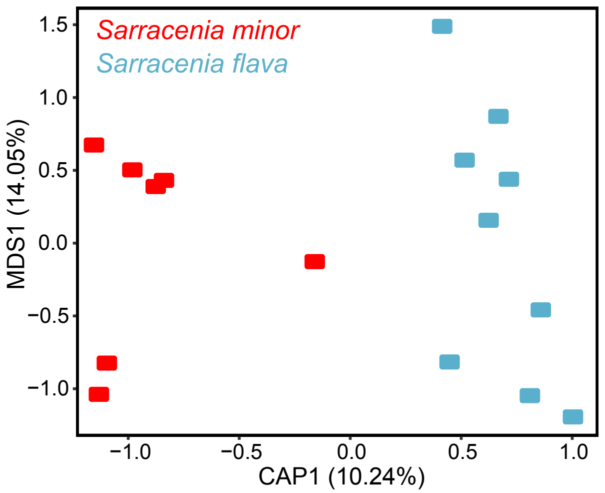

Sarracenia minor
Sarracenia flava
1.5
1.0
0.5
0.0
−0.5
−1.0
MDS1 (14.05%)
−1.0
−0.5
0.0
0.5
1.0
CAP1 (10.24%)

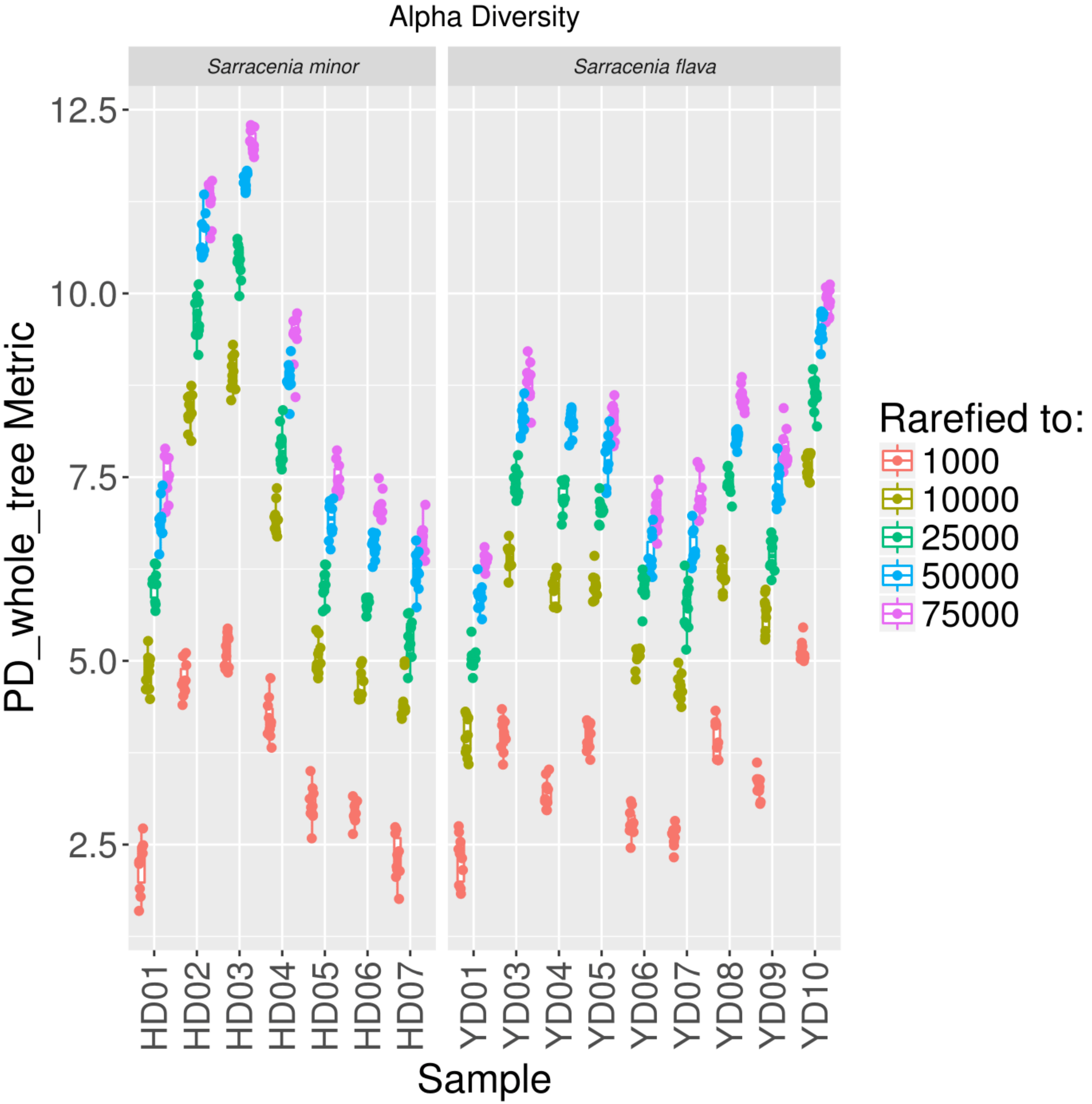
Alpha Diversity
Sarracenia minor
Sarracenia flava
12.5
10.0
7.5
5.0
2.5
PD_whole_tree Metric
HD01
HD02
HD03
HD04
HD05
HD06
HD07
YD01
YD03
YD04
YD05
YD06
YD07
YD08
YD09
YD10
Sample
Rarefied to:
1000
10000
25000
50000
75000
